# Traumatic Sternal Fractures can be Safely Treated Conservatively - A 13-Year Retrospective Cohort Study

**DOI:** 10.26502/jsr.10020170

**Published:** 2021-10-25

**Authors:** Dorine S Klei, Hilde Schutte, F Cumhur Öner, Mark CPM van Baal, Luke PH Leenen, Karlijn JP van Wessem

**Affiliations:** 1 Department of Trauma Surgery, University Medical Centre Utrecht, Utrecht, The Netherlands; 2 Department Orthopaedic Surgery, University Medical Centre Utrecht, Utrecht, The Netherlands

**Keywords:** Traumatic sternal fractures, Treatment, Outcomes, Retrospective cohort study

## Abstract

**Background:**

Traumatic sternal fractures are rare injuries with little evidence supporting the best treatment strategy. This study assessed treatment outcomes from our level-I trauma centre.

**Methods:**

A retrospective cohort study was conducted, including all sternal fracture patients admitted to our level-I trauma centre between 2007 and 2019. Patients with sternal fractures due to cardiopulmonary resuscitation, patients <16 years, patients who died during initial hospital stay, and patients lost to follow-up were excluded from analysis.

**Results:**

In 13 years, 355 patients with traumatic sternal fractures were admitted, corresponding to 2% of all trauma patients. 262 patients were included in analysis. Mean age was 52 years and 71% of patients were male. Mean ISS was 19 (range 4-66). The majority of sternal fractures was located in the sternal body. Six patients (2%) underwent primary sternal fixation. Treatment failure occurred in three patients (1%) and was significantly higher in the surgical treatment group (p=0.001). There was no difference in treatment failure between patients with and without concomitant spinal fractures.

**Conclusions:**

Conservative treatment is safe and effective for traumatic sternal fractures. Surgical treatment should be reserved for rare cases, such as imminent respiratory failure or debilitating symptomatic non-union.

## 1. Introduction

Traumatic sternal fractures are relatively uncommon injuries with an estimated incidence of 2-8% in all blunt trauma patients, contributing to less than 0,5% of all fractures [1–4]. Sternal fractures are associated with direct blunt trauma caused by seat belt or steering wheel in traffic accidents, but these injuries also frequently result from combined direct trauma and indirect flexion-compression or flexion-rotation injury of the chest, such as in high deceleration or falls from height [2,4–14]. The incidence of sternal fractures has substantially increased since the introduction of seatbelt legislation in 19836. Moreover, computed tomography (CT) scanning has become common practice in blunt trauma evaluation, probably leading to a higher detection rate, as up to 94% of sternal fractures may be missed on conventional chest radiography [15,16]. The clinical relevance of isolated sternal fractures has not been clearly established. Traditionally, sternal fractures are considered harbingers of severe concomitant injuries rather than individual entities requiring specific attention. According to literature, isolated sternal fractures are generally mild injuries and conservative therapy (mostly consisting of analgesia) suffices for most patients [17–19]. Short-term mortality and morbidity are mainly determined by concomitant injuries, including head injuries, fractures of spine and rib cage, pulmonary injuries, and cardiac injuries [6,10,12,19]. However, sternal fractures have recently gained more individual attention, accompanied by a rise in experimental surgery as well as a growing focus on long-term functional outcomes, since many patient may suffer functional limitations after sternal fractures 20-22]. Currently, no specific guidelines are available for diagnostic work-up and treatment strategies for traumatic sternal fractures [20,23]. In literature, many indications for sternal fixation have been reported: these include fracture dislocation (>1 shaft width), comminuted fractures, respiratory failure, flail chest, sternal instability, severe pain, and chest deformity. In the long term, symptomatic sternal non-union and secondary displacement might also require surgical intervention [20,24–26]. Sternal fracture treatment remains an underexposed topic. As available evidence consists mainly of case series and studies of poor quality, systematic review of literature could not yet unravel the best evidence-based treatment practice for sternal fractures [20]. The aim of this retrospective cohort study was to provide an overview of the incidence, current treatment practice, and outcomes of traumatic sternal fractures. We hypothesised that conservative sternal fracture treatment is safe and effective in most patients.

## 2. Materials and Methods

### 2.1 Study design

A retrospective single-centre cohort study was performed at the level-1 trauma centre of University Medical Centre Utrecht (UMCU), the Netherlands, from January 2007 to December 2019. Data was collected from the trauma registry maintained by the Trauma Care Network of Central Netherlands (TZNMN), which comprises all acute trauma patients admitted to our hospital. Patients with sternal fractures were identified by the Abbreviated Injury Scale (AIS) codes. Patients with traumatic sternal fractures who were examined at the emergency department within 48 hours after initial trauma and who were admitted to our hospital were included in this study. Patients with sternal fractures due to cardiopulmonary resuscitation were excluded. Patients’ demographics, injury mechanism, injury severity score, length of hospital stay, and in-hospital mortality were collected. Patients lost to follow-up (who were transferred to another hospital or did not visit the outpatient clinic after hospital discharge), patients who died during initial hospital stay, and patients under the age of 16 years were excluded from analysis. All patients received the same standard of care, consisting of a primary and secondary survey according to ATLS protocol [27], additional CT-imaging depending on clinical and X-ray findings, and hospital admission in case of multiple injuries or severe pain. Follow-up in outpatient clinic was tailored to individual patients’ needs depending on injury severity and patient preference. In case of isolated non-displaced sternal fractures, patients were instructed to only visit the outpatient clinic in case of problems. As a consequence, treatment success or failure detection in the long term depended on patients’ initiative to report complaints. To eliminate this potential bias, patients who did not visit the outpatient clinic were considered lost to follow-up, regardless of their reason for not visiting. Outpatient visits consisted of thorough clinical examination; radiological examination was at the treating physician’s discretion. Any associated spinal fractures were treated according to AOSpine guidelines; indication for surgical fixation of concomitant rib fractures was mainly guided by the inability to breath normally with adequate analgesia [28,29]. This study was approved by the institutional medical-ethical review board (METC Utrecht, WAG/mb/16/030735/WAG/mb/17/032781/WAG/mb/20/006460), and was performed in accordance with relevant guidelines and regulations.

### 2.2 Data collection

Additional data on past medical history, diagnostics, concomitant injuries, treatment, and complications were collected from individual electronic patient files. Length of follow-up was defined as the time from hospital admission to the last follow-up visit to the Surgical, Orthopaedic or Rehabilitation outpatient clinic at our hospital. Osteoporosis was defined as a positive DEXA (dual-energy X-ray absorptiometry) scan within three months post-trauma or recently started medication for osteoporosis. Sternal fractures were assigned to one of four fracture locations based on CT-scan: manubrium, manubriosternal joint (only in case of fracture-dislocations), sternal body, or xiphoid process. Sternal fracture displacement was defined as dislocation of ≥1 shaft width on lateral X-ray or sagittal CT-image. A senior orthopaedic spine surgeon (FCO) retrospectively classified all concomitant spinal fractures according to the AOSpine injury classification system [30–32]. Cardiac contusion was registered in case of diagnosis by a cardiologist. Glasgow Coma Scale (GCS) was recorded at hospital admission. Patients who were intubated and/or sedated upon arrival at the emergency department were assigned a GCS-score of 3 points. Cervical vascular injury was defined as dissection of the carotid artery or vertebral artery. Cerebral injury consisted of epidural/subdural hematoma, subarachnoid haemorrhage, or cerebral contusion. Limb and pelvic fractures were registered as extremity fractures. Primary treatment method was either conservative or operative (within 7 days post-trauma). Treatment methods were registered for sternal fractures, spinal fractures, and costal fractures. Primary outcome parameter was sternal treatment failure, defined as either surgery after failed conservative treatment or reoperation after primary surgical treatment. Treatment failure could be caused by secondary dislocation, non-union (absence of callus formation three months after trauma), technical failure (malposition of or pain due to osteosynthesis materials), or post-surgical infection. Secondary outcome parameters were hospital length of stay (H-LOS), intensive care unit length of stay (ICU-LOS), days of mechanical ventilation (DOV), wound infection, and pneumonia. Wound infection was defined as clinical signs of wound infection or positive wound culture after operative treatment. Pneumonia was defined as a positive sputum culture, pulmonary consolidations on chest radiography suspected for pneumonia, or empirical treatment for pneumonia during hospital admission.

### 2.3 Statistical analysis

Statistical analysis was performed using the R Statistical Computer Environment (an integrated development environment for statistical computing and graphics). Categorical data were presented as ratio (percentage). Significant differences were calculated through Fisher’s exact test, because of small group sizes. For continuous variables, normality of distribution was assessed by quantile-quantile (Q-Q) plots and Kernel density scores. In case of normal distribution, results were expressed as mean (range); in case of non-normal distribution, they were displayed as median [interquartile range, IQR]. Significant differences were calculated using the Student’s t-test or Mann-Whitney U-test, respectively. For all analyses, a two-sided p-value <0.05 was used as a threshold for statistical significance. A subgroup analysis was carried out for concomitant spinal fractures.

## 3. Results

**Figure 1:**
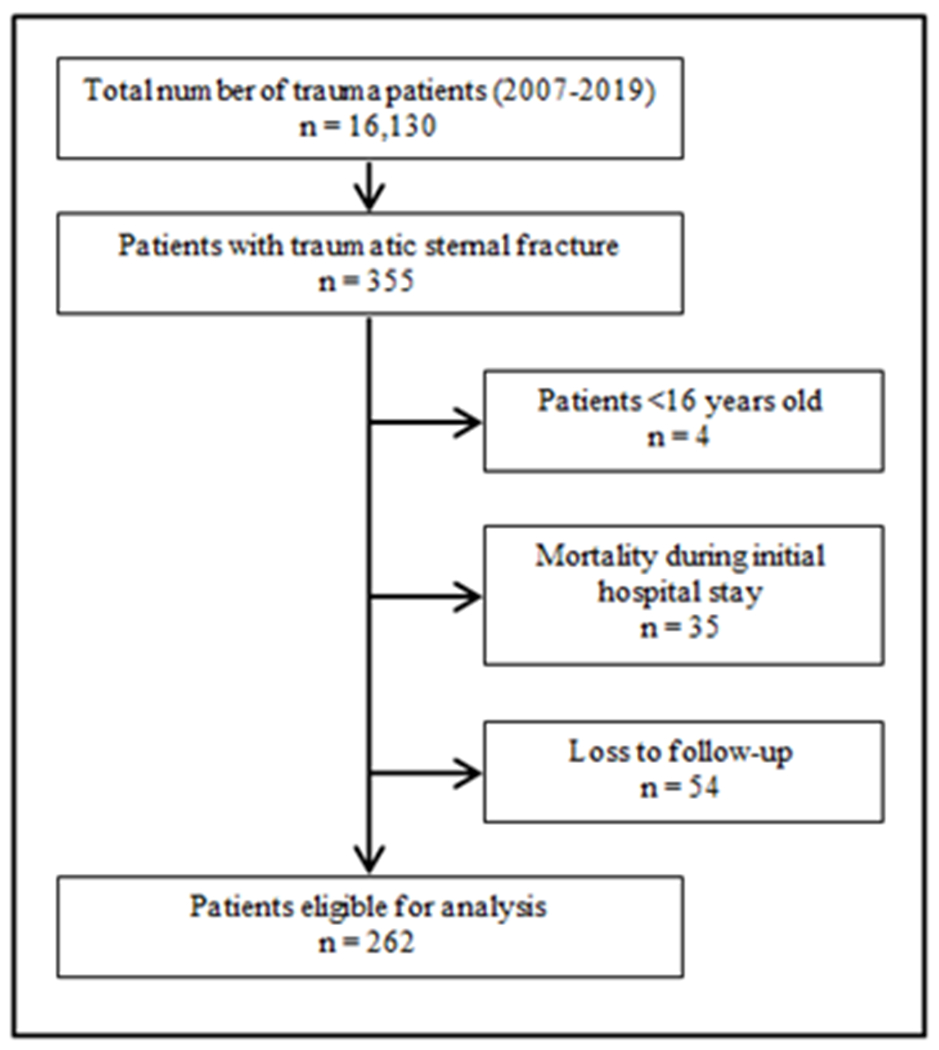
Patient flow chart.

Between January 2007 and December 2019, a total of 16,130 trauma patients were admitted to our level-1 trauma centre. In total, 355 patients suffered from a traumatic sternal fracture, corresponding to an incidence of 2%. Of these 355 patients, four patients were under the age of 16 years, 35 patients died during initial hospital stay, and 54 patients were lost to follow-up; these patients were excluded. The remaining 262 patients were included in statistical analyses (Figure 1).

### 3.1 Baseline characteristics

Of 262 patients, 185 (71%) were male. Mean age was 52 years (range 16-93 years). Five patients (2%) suffered from osteoporosis. Nineteen patients (7%) had a history of malignancy. All but one patient sustained blunt traumatic injury, caused by traffic incidents (75%), falls from >3m height (11%), falls from <3m height (10%), or other causes (3%; for instance, an animal attack or direct impact from a heavy object). One patient suffered from penetrating injury due to a gunshot wound. Mean ISS was 19 (range 4-66) (Table 1). The majority of patients (n = 240, 92%) had a single sternal fracture, while 22 patients (8%) suffered from two sternal fractures. 106 patients (40%) had a fracture located in the manubrium, 4 patients (2%) at the manubriosternal joint (MS-joint), 170 patients (65%) in the sternal body, and 4 patients (2%) in the xiphoid process. A dislocated sternal fracture was seen in eight patients (3%). Concomitant spinal fractures were present in 140 patients (53%); of these, 81 patients (58%) had a stable (AOSpine type A) fracture, while 59 patients (42%) had an unstable (AOSpine type B or C) spinal fracture. Associated thoracic injuries were seen in 212 patients (81%), most frequently rib fractures (n = 176, 67%). 27 of these patients (15%) underwent operative rib fixation (Table 1). Median GCS was 15 (IQR 14-15); traumatic brain injury occurred in mild (30 patients, 12%), moderate (22 patients, 9%), or severe form (22 patients, 9%). Notably, 19 of these 22 patients were already intubated before arrival at the emergency department and thus received a GCS-score of 3 points. Cerebral injury was diagnosed in 43 patients (16%), cervical vascular injury in 10 patients (4%), abdominal injury in 59 patients (23%), and extremity injury in 122 patients (47%). Median follow-up duration was 39 weeks (IQR 10-88 weeks) (Table 1).

### 3.2 Treatment methods

256 patients (98%) received conservative treatment for their sternal fractures. Six patients (2%) underwent primary sternal fixation. Indications for sternal fixation were severe pain (n = 1), dislocation ≥1 shaft width (n = 1), fracture-dislocation at the manubriosternal joint (n = 1), and flail chest (n = 3). All operations took place from 2010 to 2014 (Table 2). In the operative sternal treatment group, all five patients with concomitant rib fractures underwent surgical rib fixation, while in the conservative sternal treatment group, only 22 patients (13%) underwent operative rib treatment (p<0.001). At baseline, there were no other differences between the conservative and operative sternal treatment groups (Table 1).

### 3.3 Treatment outcomes

**Figure 2:**
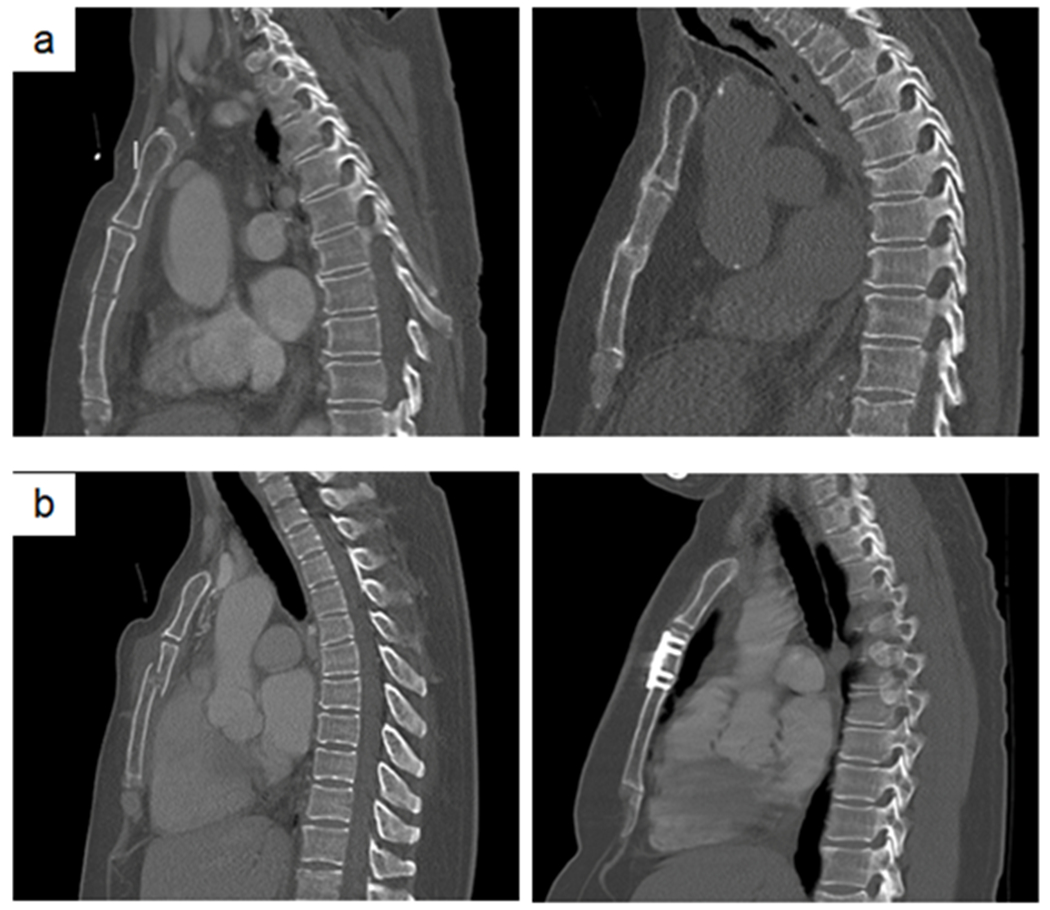
Chest CT images of traumatic sternal fractures. a- Conservatively treated sternal fracture in a 74-year old female patient, at trauma (left) and at follow-up after seven years (right). b- Surgically treated sternal fracture in a 50-year old male patient, at trauma (left) and at follow-up after two years (right).

Treatment failure occurred in three patients (1%), whereby significantly more patients in the operative group showed treatment failure (33% vs. 0.4%, p<0.001). One patient in the conservative treatment group underwent secondary surgery because of secondary dislocation. Two patients in the operative treatment group required re-operation due to wound infection. Median length of hospital stay was 11 days (IQR 7-22 days). 83 patients (32%) were admitted to ICU, with a median stay of eight days (IQR 4-16 days) with five days of mechanical ventilation (IQR 2-13 days); readmission to ICU occurred in eight patients (10%). Two out of six patients (33%) who underwent primary surgery developed a postoperative sternal wound infection; these were the patients who underwent re-operation. 51 patients (19%) suffered from pneumonia. There were no significant differences in secondary outcome parameters between the conservative and operative sternal treatment groups (Table 3). Chest CT images of conservative and surgical sternal treatment, at baseline and at follow-up, are presented in Figure 2.

### 3.4 Subgroup analysis of concomitant spinal fractures

Of 262 patients, 140 patients (53%) had a concomitant spinal fracture. Spinal fractures were AOSpine type A (n = 81, 58%), type B (n = 49, 35%), or type C (n = 10, 7%). These patients were more severely injured (ISS 23 vs. 15, p<0.001) and injuries were more frequently caused by falls from >3m height (18% vs 2%, p<0.001). Furthermore, these patients more often had sternal fractures located in the manubrium (49% vs 31%, p= 0.005) and showed a higher incidence of several thoracic injuries such as rib fractures (Table 4). Two patients with concomitant spinal fractures (1%) and four patients without spinal fracture (3%) underwent sternal fixation. There was no significant difference in sternal treatment failure. Patients with concomitant spinal fractures were more often admitted to ICU (39% vs 24%, p= 0.012), stayed longer in ICU (9 vs. 5 days, p= 0.011) and in hospital (14 vs. 8 days, p<0.001), and more often developed pneumonia (30% vs. 7%, p<0.001) (Table 5).

## 4. Discussion

In the present cohort, an appropriate restraint towards surgical intervention was observed. Only six out of 262 patients were treated surgically. Treatment failure occurred in only three patients (1%) and was significantly lower in the conservative treatment group: failure occurred in one patient (0.4%) in the conservative treatment group, but in two patients (33%) in the surgical treatment group. These findings support our recommendation that sternal fractures should be managed conservatively. However, these results must be interpreted with caution. On the one hand, treatment failure might be underestimated in the conservative treatment group since one patient showed asymptomatic non-union, but conservative treatment was continued. Therefore, this patient did not meet our definition of treatment failure. On the other hand, treatment failure might be overestimated in the operative treatment group, because two patients who needed re-operation due to postoperative wound infection (one patient requiring removal of osteosynthesis material) were counted as treatment failure. One could question whether the latter cases should be regarded as genuine treatment failure. Moreover, the operative sternal treatment group was small; hence, results for this treatment group might be skewed. Interestingly, in the surgical treatment group, patients with concomitant rib fractures (5 out of 6 patients) underwent fixation of both sternum and ribs. These patients had extensive rib fractures with flail chest, worsened by the presence of a (dislocated) sternal fracture. Hence, fixation of both rib and sternum was performed. Most importantly, all but one patient with treatment failure eventually showed satisfactory treatment outcomes. The two surgically treated patients suffering from wound infection reported good functional outcome during their last outpatient visit; one of them showed radiologic consolidation, while the other showed consolidation of one of two sternal fractures (the other non-union being non-symptomatic). The patient with symptomatic sternal non-union (treated conservatively) also suffered from a type B spinal fracture which required surgical fixation; this patient reported mild remaining back problems. The patient with secondary sternal dislocation, however, required multiple re-operations for both sternal and spinal fractures and to date, has not been able to resume his work or hobbies. Previous studies, mostly consisting of case reports and small case series, reported good outcomes of both conservative and operative sternal treatment [20,23,24]. Two recent propensity-matched studies by Christian et al. [23] and Choi et al. [33], surgically treated patients had a longer hospital stay, but lower mortality rate than conservatively treated patients; pulmonary complications occurred at the same rate in both treatment groups. Fracture healing and functional outcomes were, however, not reported [23,33]. In the present cohort study, 98% of patients were treated conservatively with good outcome, indicating that conservative treatment is safe and effective for almost all sternal fractures. Only six patients underwent primary surgery, of whom two patients developed a wound infection. Based on these findings, sternal fractures should be initially treated conservatively. Surgical treatment of sternal fractures should be reserved for imminent respiratory failure due to severe fracture dislocation or flail chest, or severe symptomatic non-union. Surgical indications should be standardised and set carefully by an experienced team, with potential complications in mind. Notably, improved analgesic techniques could play an important role in reducing pulmonary morbidity due to severe pain and thereby avert surgical treatment in the acute setting [34–36]. Biomechanically, based on the four-column spine model, combined sternovertebral fractures could result in severe thoracic wall instability [37]. In various studies, concomitant spinal fractures are considered an indication for sternal fixation [7,38]. In the current cohort, sternal fixation was performed at similar rates in patients with and without concomitant spinal fractures, whereby the presence of spinal injury in itself was not sufficient to perform sternal fixation. According to AOSpine guidelines, almost all type B/C spinal fractures were fixated or treated with haloframe. Subgroup analysis showed that sternal treatment failure did not differ significantly between patients with and without concomitant spinal fractures. These results support our previous finding that there is no general indication for sternal fixation in sternovertebral fracture patients1. Patients in the spinal fracture group had a significantly higher ISS. Consequently, secondary treatment outcomes for patients with concomitant spinal fractures were worse than patients without a spinal fracture and were not directly related to sternovertebral injuries themselves. Fifty patients (19%) had an associated cardiac contusion. Cardiac contusion is a serious and potentially lethal complication of blunt chest trauma [2,4,14,39–41]. Currently, a clear definition of cardiac contusion is lacking, as well as a gold standard for diagnostic tests. Therefore, management is based on diagnostic findings rather than on the diagnosis of cardiac contusion [39]. In the present study, cardiac contusion was diagnosed only after confirmation by a cardiologist, based on electrocardiogram (ECG), biomarkers (troponin), and/or transthoracic echocardiogram (TTE) findings. However, diagnostic procedures considerably varied per patient. In future studies we will focus on cardiac contusion in sternal fracture patients, as clear definitions and guidelines are crucial to avoid over-diagnosis and over-treatment of this injury. To our knowledge, the present study comprises the largest patient cohort to date for which treatment outcomes are reported. With a median follow-up duration of 39 weeks, both short-term and long-term treatment complications could be detected. Using a prospective trauma registry, a complete cohort of patients was included in our cohort study, largely making up for the risk of information and reporting bias in a retrospective single-centre study design. Nonetheless, several limitations apply to this study. Notably, 54 patients of our cohort were lost to follow-up: 23 patients were transferred to other hospitals and 31 patients did not visit our outpatient clinic. However, many of these patients were advised to visit the outpatient clinic only in case of problems; it could thus be assumed that these patients had an adequate recovery. In addition, since the surgical treatment group was small, statistical outcomes might be skewed. Moreover, years after date, clinical decisions regarding type and timing of sternal treatment could not be reported in great detail. Lastly, as highlighted by the large number of patients with associated spinal fractures (53%) and rib fractures (67%), these concomitant thoracic fractures could play a role in thoracic wall instability and, hence, in sternal consolidation. However, since 85% of patients with concomitant rib fractures were treated conservatively for their rib fractures as well, the role of these associated injuries will likely not have been an important confounder. In addition, due to the relatively small patient population and the limited surgical sternal treatment group, correction for these factors was considered unfeasible. The present study rather aimed to present general sternal treatment outcomes, which are severely underexposed in literature [20].

## 5. Conclusion

In conclusion, conservative treatment with adequate analgesia is preferable for almost all patients with a sternal fracture. Surgical treatment inherently bears the risk of complications such as wound infections, and should be reserved for rare indications, such as imminent respiratory failure or severe symptomatic non-union.

## Figures and Tables

**Table 1:** Baseline characteristics^a^

	Overall (n = 262)	Conservative sternal treatment (n = 256)	Operative sternal treatment (n = 6)	p-value
**Patient characteristics**				
Age (mean (range))	52 (16-93)	52 (16-93)	56 (45-73)	0.563
Male (%)	185 (71)	181 (71)	4 (67)	1
Osteoporosis (%)	5 (2)	5 (2)	0	1
History of malignancy (%)	19 (7)	19 (7)	0	1
Trauma type (%)				1
Blunt trauma	261 (100)	255 (100)	6 (100)	
Penetrating trauma	1 (0)	1 (0)	0	
Blunt trauma mechanism (%)				1
Traffic accident	197 (75)	191 (75)	6 (100)	
Fall from >3m height	28 (11)	28 (11)	0	
Fall from ≤3m height	27 (10)	27 (11)	0	
Other	9 (3)	9 (4)	0	
ISS (mean (range))	19 (4-66)	19 (4-66)	18 (4-38)	0.788
**Sternal fracture characteristics**				
Number of sternal fractures (%)				0.082
1 fracture	240 (92)	236 (92)	4 (67)	
2 fractures	22 (8)	20 (8)	2 (33)	
Sternal fracture location (%)^b^				
Manubrium	106 (40)	105 (41)	1 (17)	0.406
Manubriosternal joint	4 (2)	3 (1)	1 (17)	0.089
Sternal body	170 (65)	164 (64)	6 (100)	0.094
Xiphoid process	4 (2)	4 (2)	0	1
Dislocation of sternal fracture (%)	8 (3)	7 (3)	1 (17)	0.171
**Associated injuries**				
Associated spinal fractures (%)	140 (53)	138 (54)	2 (33)	0.422
AOSpine classification (%)^c^				0.592
Type A	81 (58)	79 (57)	2 (100)	
Type B	49 (35)	49 (36)	0	
Type C	10 (7)	10 (7)	0	
Associated thoracic injuries (%)	212 (81)	207 (81)	5 (83)	1
Rib fracture	176 (67)	171 (67)	5 (83)	0.667
Primary rib treatment^d^				<0.001*
Conservative rib treatment	149 (85)	149 (87)	0	
Operative rib treatment	27 (15)	22 (13)	5 (100)	
Clavicular fracture	45 (17)	44 (17)	1 (17)	1
Lung contusion	105 (40)	102 (40)	3 (50)	0.686
Pneumothorax	94 (36)	93 (36)	1 (17)	0.425
Hematothorax	23 (9)	23 (9)	0	1
Cardiac contusion	50 (19)	49 (19)	1 (17)	1
Other thoracic injuries	62 (24)	59 (23)	3 (50)	0.147
Other associated injuries (%)				
GCS (median [IQR])	15 [14-15]	15 [14-15]	15 [14-15]	0.978
Mild TBI (GCS 13-14)	30 (12)	28 (11)	2 (33)	0.149
Moderate TBI (GCS 9-12)	22 (9)	22 (9)	0	1
Severe TBI (GCS ≤8)	22 (9)	22 (9)	0	1
Cerebral injury	43 (16)	43 (17)	0	0.593
Cervical vascular injury	10 (4)	10 (4)	0	1
Abdominal injury	59 (23)	58 (23)	1 (17)	1
Extremity injury	122 (47)	119 (46)	3 (50)	1
**Follow-up**				
Follow-up in weeks (median [IQR])	39 [10-88]	38 [10-87]	54 [24-86]	0.563

Abbreviations: ISS, injury severity score; GCS, Glasgow Coma Scale; TBI, traumatic brain injury; IQR, interquartile range.

a Due to rounding off, percentages might not add up to 100%.

b Sternal fracture location is displayed as the percentage of patients with a sternal fracture in a particular location. 22 patients had 2 sternal fractures and were counted in 2 groups.

c Percentage was calculated based on the number of patients with a spinal fracture.

d Percentage was calculated based on the number of patients with a rib fracture.

e 19 of these 22 patients were intubated before arrival at the emergency department and received a GCS-score of 3 points.

* Statistically significant difference (p<0.05).

**Table 2:** Incidence and treatment of sternal fractures

Year	Total number of trauma patients	Patients with sternal fracture	Primary sternal fixation
2007	771	7	-
2008	863	11	-
2009	1090	22	-
2010	1315	24	3
2011	1313	16	-
2012	1349	21	1
2013	1280	13	1
2014	1372	20	1
2015	1348	19	-
2016	1419	30	-
2017	1453	25	-
2018	1342	25	-
2019	1215	29	-

**Table 3:** Treatment methods and outcomes^a^

	Overall (n = 262)	Conservative sternal treatment (n = 256)	Operative sternal treatment (n = 6)	p-value
**Sternal fracture treatment**				
Sternal treatment failure (%)	3 (1)	1 (0.4)	2 (33)	0.001*
Infection	2 (67)	0	2 (100)	
Non-union	0	0	0	
Secondary dislocation	1 (33)	1 (100)	0	
**Secondary outcome parameters**				
Hospital LOS in days (median [IQR])	11 [7-22]	11 [7-23]	14 [10-17]	0.874
ICU				
Admission to ICU (%)	83 (32)	81 (32)	2 (33)	1
ICU LOS in days (median [IQR])	8 [4-16]	8 [3-16]	6 [6-6]	0.613
DOV (median [IQR])	5 [2-13]	5 [2-13]	4 [4-5]	0.776
Readmission to ICU (%)^b^	8 (10)	8 (10)	0	1
Sternal wound infection (%)^c^	2 (29)	0	2 (33)	1
Pneumonia (%)	51 (19)	51 (20)	0	0.6

Abbreviations: LOS, length of stay; ICU, intensive care unit; DOV, days of mechanical ventilation; IQR, interquartile range.

a Due to rounding off, percentages might not add up to 100%.

b Percentage was calculated based on the number of patients who were admitted to ICU.

c Percentage was calculated based on the number of patients who underwent sternal fixation (total n=7; primary sternal fixation n=6; secondary operation n=1).

* Statistically significant difference (p<0.05).

**Table 4:** Baseline characteristics stratified for spinal fractures^a^

	Overall (n = 262)	Spinal fracture (n = 140)	No spinal fracture (n = 122)	p-value
**Patient Characteristics**				
Age (mean (range))	52 (16-93)	51 (16-93)	52 (17-92)	0.789
Male (%)	185 (71)	99 (71)	86 (70)	1
Osteoporosis (%)	5 (2)	2 (1)	3 (2)	0.666
History of malignancy (%)	19 (7)	10 (7)	9 (7)	1
Trauma type (%)				0.466
Blunt trauma	261 (100)	140 (100)	121 (99)	
Penetrating trauma	1 (0)	0	1 (1)	
Blunt trauma mechanism (%)				<0.001*
Traffic accident	197 (75)	90 (64)	107 (88)	
Fall from >3m height	28 (11)	26 (19)	2 (2)	
Fall from ≤3m height	27 (10)	20 (14)	7 (6)	
Other	9 (3)	4 (3)	5 (4)	
ISS (mean (range))	19 (4-66)	23 (4-66)	15 (4-50)	<0.001*
**Sternal fracture characteristics**				
Number of sternal fractures (%)				0.074
1 fracture	240 (92)	124 (89)	116 (95)	
2 fractures	22 (8)	16 (11)	6 (5)	
Sternal fracture location (%)^b^				
Manubrium	106 (40)	68 (49)	38 (31)	0.005*
Manubriosternal joint	4 (2)	3 (2)	1 (1)	0.626
Sternal body	170 (65)	84 (60)	86 (70)	0.092
Xiphoid process	4 (2)	1 (1)	3 (2)	0.341
Dislocation of sternal fracture (%)	8 (3)	3 (2)	5 (4)	0.479
**Associated injuries**				
Associated spinal fractures				
AOSpine classification (%)^c^				
Type A	81 (58)	81 (58)	-	
Type B	49 (35)	49 (35)	-	
Type C	10 (7)	10 (7)	-	
Associated thoracic injuries (%)	212 (81)	118 (84)	94 (77)	0.157
Rib fracture	176 (67)	104 (74)	72 (59)	0.012*
Primary rib treatment^d^				1
Conservative rib treatment	149 (85)	88 (85)	61 (85)	
Operative rib treatment	27 (15)	16 (15)	11 (15)	
Clavicular fracture	45 (17)	28 (20)	17 (14)	0.25
Lung contusion	105 (40)	63 (45)	42 (34)	0.1
Pneumothorax	94 (36)	60 (43)	34 (28)	0.014*
Hematothorax	23 (9)	18 (13)	5 (4)	0.015*
Cardiac contusion	50 (19)	25 (18)	25 (20)	0.638
Other thoracic injuries	62 (24)	34 (24)	28 (23)	0.884
Other associated injuries (%)				
GCS (median [IQR])	15 [14-15]	15 [14-15]	15 [15-15]	0.149
Mild TBI (GCS 13-14)	30 (12)	18 (13)	12 (10)	0.56
Moderate TBI (GCS 9-12)	22 (9)	14 (10)	8 (7)	0.377
Severe TBI (GCS ≤8)	22 (9)	13 (9)	9 (8)	0.659
Cerebral injury	43 (16)	25 (18)	18 (15)	0.51
Cervical vascular injury	10 (4)	8 (6)	2 (2)	0.111
Abdominal injury	59 (23)	35 (25)	24 (20)	0.374
Extremity injury	122 (47)	68 (49)	54 (44)	0.535
**Follow-up**				
Follow-up in weeks (median [IQR])	39 [10-88]	52 [27, 99]	15 [6, 60]	<0.001*

Abbreviations: ISS, injury severity score; GCS, Glasgow Coma Scale; TBI, traumatic brain injury; IQR, interquartile range.

a Due to rounding off, percentages might not add up to 100%.

b Sternal fracture location is displayed as the percentage of patients with a sternal fracture in a particular location. 22 patients had 2 sternal fractures and were counted in 2 groups.

c Percentage was calculated based on the number of patients with a spinal fracture.

d Percentage was calculated based on the number of patients with a rib fracture.

e 19 of these 22 patients were intubated before arrival at the emergency department and received a GCS-score of 3 points.

* Statistically significant difference (p<0.05).

**Table 5:** Treatment methods and outcomes stratified for spinal fractures^a^

	Overall (n = 262)	Spinal fracture (n = 140)	No spinal fracture (n = 122)	p-value
**Sternal fracture treatment**				
Primary sternal treatment (%)				0.422
Conservative treatment	256 (98)	138 (99)	118 (97)	
Operative treatment	6 (2)	2 (1)	4 (3)	
Sternal treatment failure (%)	3 (1)	1 (1)	2 (2)	0.599
Infection	2 (67)	0	2 (100)	
Non-union	0	0	0	
Secondary dislocation	1 (33)	1 (100)	0	
**Secondary outcome parameters**				
Hospital LOS in days (median [IQR])	11 [7-22]	14 [8-28]	8 [5-19]	<0.001*
ICU				
Admission to ICU (%)	83 (32)	54 (39)	29 (24)	0.012*
ICU LOS in days (median [IQR])	8 [4-16]	9 [4-17]	5 [2-10]	0.011*
DOV (median [IQR])	5 [2-13]	7 [3-14]	4 [1-7]	0.06
Readmission to ICU (%)^b^	8 (10)	5 (9)	3 (10)	1
Sternal wound infection (%)^c^	2 (29)	0	2 (50)	0.429
Pneumonia (%)	51 (19)	42 (30)	9 (7)	<0.001*

Abbreviations: LOS, length of stay; ICU, intensive care unit; DOV, days of mechanical ventilation; IQR, interquartile range.

a Due to rounding off, percentages might not add up to 100%.

b Percentage was calculated based on the number of patients who were admitted to ICU.

c Percentage was calculated based on the number of patients who underwent sternal fixation (total n=7; primary sternal fixation n=6; secondary operation n=1).

* Statistically significant difference (p<0.05).
